# The system of institutional care for children with developmental disabilities in Russia: problems and current results of reforming

**DOI:** 10.1192/j.eurpsy.2022.1077

**Published:** 2022-09-01

**Authors:** O. Rusakovskaya, E. Volodenkova

**Affiliations:** 1 V. Serbsky National Medical Research Centre for Psychiatry and Narcology, Forensic Psychiatry In Civil Process, Moscow, Russian Federation; 2 Krasnoyarsk Region Neuropsychiatric Facility, Ministry Of Health, Krasnoyarsk, Russian Federation

**Keywords:** institutional care, Child Psychiatry, intellectual disability, WISC

## Abstract

**Introduction:**

As part of the full-sized personal examination of persons, living in residential facilities for mentally disabled people (Kekelidze, 2020) 621 children, living in 3 asylums “for children with mental retardation” of Krasnoyarsk region were examined. In 134 cases diagnosis “Moderate or severe mental retardation” wasn’t confirmed. In 2020 a full examination of 47 children with non-confirmed diagnoses was carried out in one of the asylums.

**Objectives:**

The main purpose of this investigation is to present the results of this full examination and sum up the main problems of the system of institutional care for children with intellectual and developmental disabilities in Russia.

**Methods:**

Clinical and psychological examination, Wechsler Intelligence Scale for Children (WISC-II), analysis of pedagogical characteristics.

**Results:**

Figure 1 presents the results of cluster analysis of WISC-II. In 37 cases diagnoses were reversed to Mild intellectual disability. In one case – 10 y.o. boy - to developmental delay through social-pedagogic neglect.

**Figure:**
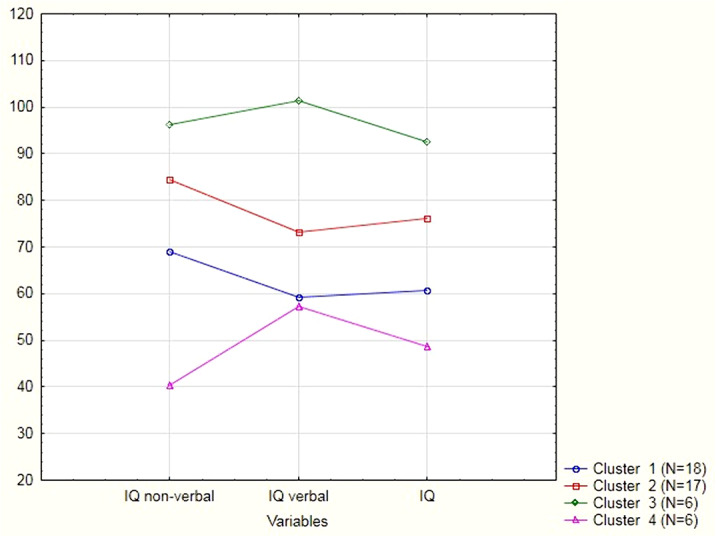

In the beginning of 2021 32 children were transferred to boarding schools with educational programme for children with mild intellectual disabilities. 13 children adapted to new developmental and educational conditions relatively successfully, 4 children – unsuccessfully and were returned to the first institute.

**Conclusions:**

The success of adaptation did not depend on IQ, but on the age of the child and the severity of emotional and behavioral disorders, as well as on the willingness of the institution to provide personalized assistance to the child. The results identify the main problems of the system of institutional care for children with developmental disabilities in Russia.

**Disclosure:**

No significant relationships.

